# The First Finding of Six Instars of Larvae in Heteroptera and the Negative Correlation between Precipitation and Number of Individuals Collected in Sea Skaters of *Halobates* (Heteroptera: Gerridae)

**DOI:** 10.3390/insects7040073

**Published:** 2016-12-07

**Authors:** Tetsuo Harada, Takahiro Furuki, Wataru Ohoka, Noritomo Umamoto, Mitsuru Nakajo, Chihiro Katagiri

**Affiliations:** 1 Laboratory of Environmental Physiology, Graduate School of Integrated Sciences and Arts, Kochi University, Kochi 780-8520, Japan; B15m6b21@s.kochi-u.ac.jp (T.F.); b13m6b17@s.kochi-u.ac.jp (N.U.); 2 Graduate School of Medicine, Kyoto University, Kyoto 606-8501, Japan; wakamura.tomoko.5v@kyoto-u.ac.jp; 3 Laboratory of Science Education, Graduate School of Integrated Sciences and Arts, Kochi University, Kochi 780-8520, Japan; Mit-na@kochi-u.ac.jp; 4 Faculty of General Education, Tokyo Denki University, Tokyo 120-8551, Japan; 20445@ms.dendai.ac.jp

**Keywords:** sea skaters, near Sumatra, undescribed species, six instars as larval stages, negative correlation between precipitation and number of sea skaters

## Abstract

This study, conducted during a scientific cruise, MR15-04, aims, first, to examine species and larval/adult components of *Halobates* (Heteroptera: Gerridae) inhabiting the tropical Indian Ocean of 4°00′ S–7°00′ S, 101°00′ E–103°00′ E and, second, to examine the correlative relationship between precipitation just before collection and the number of sea skaters collected in November and December 2015. Near Sumatra (50 km south-west), larvae and adults of four species of *Halobates* (*Halobates germanes* White, 1883; *Halobates micans* Eschscholtz, 1822; *Halobates princeps* White, 1883; undescribed species: *Halobates* sp.) were collected. Adults of an undescribed species had about a 5 mm long body in a gourd-like shape. One male adult specimen of *H. princeps* was collected. Body length, body width, and head width was measured in all specimens of *Halobates*. Six larval stages were detected in all three species of sea skaters as the first finding for Heteropteran insects. There was a negative correlation between amount of precipitation for 19 h before collection and the number of *Halobates* individuals collected by the neuston net. Death or (positive or passive) sinking by sea skaters could be due to occasional rain fall on the sea surface.

## 1. Introduction

Many great voyages have been launched to explore the oceans and what lies beyond, as they have always held a great fascination for us. A great variety of marine organisms were collected and described during these voyages, but insects appear to have received little attention [1]. Although they are the most abundant animals on land, insects are relatively rare in marine environments [2]. However, a few thousand insect species belonging to more than 20 orders are considered to be marine [3,4]. The majority of marine insects belong to the Coleoptera, Hemiptera, and Diptera orders, and they can be found in various marine habitats. However, the only insects to live in the open ocean are members of the genus *Halobates*, commonly known as sea-skaters [2]. They belong to the family Gerridae (Heteroptera), which comprises the common pond-skaters or water-striders. Unlike most of its freshwater relatives, the genus *Halobates* is almost exclusively marine. Adults are small, measuring only about 0.5 cm in body length, but they have rather long legs and may have a leg span of 1.0–1.5 cm [1]. Although the key to identifying the species in the genus *Halobates* hase shown to be morphological details of the genitalia in adult males, as noted in the appendix of Andersen and Chen [1], the larvae have not yet been described. The primary purpose of this study is to measure the body length, body width and head width of all *Halobates* larvae collected in this cruise and clarify the number of stages of sea skater larvae collected.

Sampling of *Halobates* has been performed in different geographical positions in cruises in the Pacific Ocean. However, no information has been presented on the species dynamics and individual composition in the field near the shores of the Indonesian islands. The second purpose of this study is to clarify the species component and population density of sea skaters inhabiting the area near Sumatra.

The relationship between weather conditions such as surface water temperature and population density of sea skaters has been reported. For example, the most appropriate temperature for *Halobates micans* and *H. germanus* were around 27 to 30 °C, but ranged more widely for the smaller species of *H. sericeus*, from 22 to 30 °C [5,6]. This preference of *H. sericeus* for a wider temperature range may be related to the wider range of latitudes this species inhabits. However, the possibility that rain fall is also related to the temperature range inhabited by sea skaters has not yet been examined. The third purpose of this study is to clarify this relationship in the sea skaters inhabiting the area 50 km from Sumatra. 

## 2. Materials and Methods

### 2.1. Samplings

Samplings were performed every three days from 20 November to 14 December 2015 in the area of 4° S–7° S, 101° E–103° E with a neuston net (6 m long and diameter of 1.3 m). The neuston net was trailed for 15 mm × 3 times (3 trials) per one night on the starboard side of R/V MIRAI (8687t) owned by the Japan Agency for Marine-earth Science and TECHnology (JAMSTEC, Yokosuka, Japan). Each trailing comprised three 15-min trials at night for nine nights with the ship speed of 2.0 knot to the sea water. This was repeated twice at each station. The surface area swept by the neuston net was evaluated as an expression of (flow-meter value × 1.3 m of width of the neuston net). Precipitation was measured with radio detecting and ranging during the ship of MR15-04 on the deck of the R/V MIRAI.

### 2.2. Treatments of Specimens after the Samplings

Sea skaters trapped in the grey plastic bottle fixed to the end of the neuston net were paralyzed from the physical shock of the trailing of the net. Paralyzed sea skaters were transferred to a paper towel to respire. Paralysis subsided within 20 min. for most specimens. When sea skaters were trapped in the jelly of a jelly fish, the jelly was removed from the body very carefully and quickly by hand for recovery from paralysis.

### 2.3. Measurement of Body Sizes

Body length, body width, and head width were measured in all sea skater specimens collected during the cruise with a stereoscopic microscope, stage micrometer, and ocular micrometer. Photos of adults and larvae (*H. germanus*, *H. micans*, *H. princeps* and *H.* sp.) were taken by digital video camera (HC-V100M Panasonic; Panasonic Co Ltd., Osaka, Japan) during the cruise.

### 2.4. Statistic Analysis

The data was analyzed with SPSS 12.0 (12.0 J for Windows; SPSS Inc., Chicago, IL, USA) statistical software. ANCOVA and ANOVA analyses were performed on the relationship between body length, body width, and head width, species, and stage for the three species of *Halobates*. Body size was compared between *Halobates micans* and *Halobates* sp. with Mann-Whitney U tests for continuous values. Pearson’s correlation analysis was used for the correlative analysis between precipitation and density of sea skater specimens collected.

## 3. Results

### 3.1. Distribution

**Figure 1:**
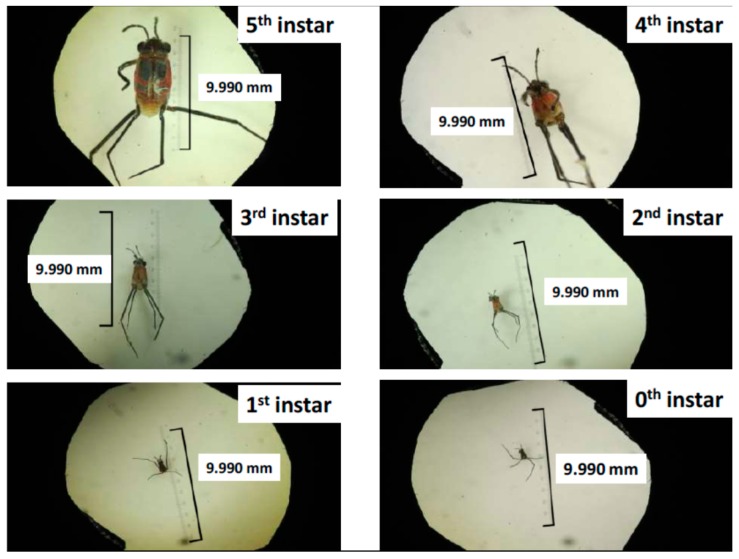
Larvae from 0th to 5th instar of *Halobates germanus* EW: Eye width, BW: Body width, BL: Body length, measured with a binocular microscope.

**Figure 2:**
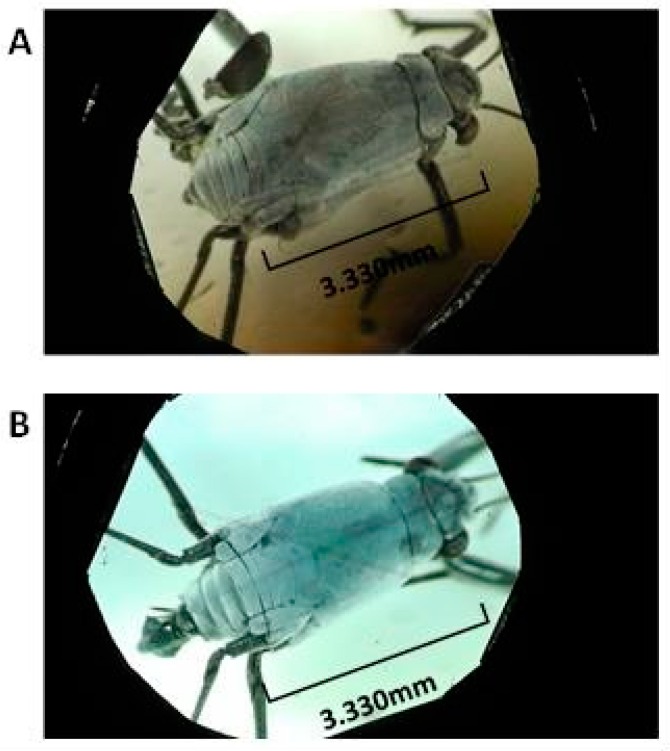
Photo from dorsal side of an un-described species, *Halobates* sp. (**A**: female, **B**: male).

In samplings of *Halobates* (Table 1 (A-2)) inhabiting tropical stations in the eastern Indian Ocean, 12 to 330 individuals were collected per trial of four species of *Halobates germanus*, *H. micans*, *H. princeps* and one undescribed and relatively large species of *H.* sp. This undescribed species has an adult body length of about 5 cm with a gourd-like shape (Figure 1 and Figure 2). Morphological study and precise comparison with all the 71 species described in the appendix of the key of the identification of *Halobates* Eschsholtz [1] indicates that this is likely a new species. Larvae and adult specimens of these four species were collected at the stations within 04°00′ S–06°00′ S, 101°00′ E–103°00′ E. The population density at Station 1 (Table 1 (A-1,2)) was moderate at about 6000 individuals/km^2^ and exclusively *H. germanus*.

At fixed stations (Stations 2–9: 04°02′ S 101°53′ E) located about 50 km in the southern-western direction from the shore of Sumatra, Indonesia, various species of *Halobates* (*H. germanus*, *H. micans*, *H. princeps* and *H.* sp.) were collected, although *H. germanus* was also dominant there. The number of individuals collected varied greatly from 12 to 327 individuals. These results imply that sea skaters are gregarious rather than spreading out on the sea surface in the tropical ocean. On average, the population density of the dominant species, *H. gerumanus* and *H.* sp. was about 20,000 and 2500, respectively, at the fixed stations (Stations 2–9 in Table 1 (A-2) and Table 2). At the Stations 6 and 7, 50 and 152 larvae were collected, respectively, and 51 exuviae (wasted skin at molting) were caught in total. Reproductive and growth activity might be active at these two stations. 

### 3.2. Body Sizes

Table 3 shows the body length, body width and head width of all larvae and adults of four species, *Halobates micans*, *H. germanus*, *H. princeps* and *H.* sp. Six instars in the larval stage were detected as the first finding of all three species (Table 3 and Figure 1). Similar to previous studies [1], all stages of larvae and adults of *H. micans* had a larger body length, body width, and head width than *H. germanus* (Table 3 and Table 4). The undescribed species had a specific gourd-like shape and the body lengths of the 2nd to 5th instars larvae and adults were significantly greater than those of the other species (for example *H. micans*) (Table 5).

## 4. Discussion

### 4.1. Distribution

Reviewing the results of seven sea skater samplings performed in the tropical to subtropical Pacific Ocean and tropical Indian Ocean, *Halobates micans* were exclusively dominant in the tropical Indian Ocean (Table 1 (B, C), and KH-10-05 cruise: Harada et al. [13]).

In the tropical Pacific Ocean in the lower latitude area of 10° S–10° N, not *H. micans* but *H. germanus* occupied the area near the islands even in the lower latitude area (Table 1 (D-2, G)) [1]. In the higher latitude area of 24°–25° N, *H. sericeus* was the exclusively dominant species in the subtropical Pacific Ocean (Table 1 (D-3, E-1, F-2)).

The difference in the species component of oceanic sea skaters due to the latitude and distance from the islands may be related to cannibalism by a bigger species (*H. micans*) of a smaller one (*H. sericeus*) (Harada, unpublished), tolerance to variation in surface temperature [1,14,15] (*H. sericeus* inhabiting a wider range of latitudes and with harder tolerance to temperature change) and salinity [16] (*H. germanus* with a harder tolerance to lower salinity because of selection by heavy rain fall from inhabiting the area near the shore).

### 4.2. Larval Development of Larvae of Sea Skaters

Most of the species included in Heteroptera, Insecta have five stages of larvae, for example the chinch bug, *Blissusleu copterus*, and the harlequin bug, *Murgantia histrionic* [17]. The measurements of body length, body width, and head width were performed in all larvae and adults collected in this cruise (MR15-04) by the R/V MIRAI in November–December 2016. This measurement made it clear that the number of larvae was not five but six because of the existence of 0th instars before the normal five instars. The cruise of MR06-04 by the R/V MIRAI was performed in the western tropical Pacific Ocean. More than 3000 eggs that had been laid on a piece of styrene foam were collected by an ORI (Ocean Research Institute of the University of Tokyo produced) net during this cruise [14]. These eggs were incubated and most 0th instar larvae hatched from the eggs [18]. However, none molted out into the next 1st instar larvae, despite careful incubation with an appropriate temperature of around 30 °C and sufficient food (larvae fish collected by the ORI and neuston nets and adult Calliphoridae flies, *Lucillia irrustris*). At least larvae of the 3rd to 5th instars had been observed to molt into the next stage during incubation in the 9 cruises by the R/V MIRAI [18]. The molt from 0th instar to 1st instar was not successful during incubation on the ships previously [19]. Specific conditions including food, temperature, PH, and lighting may be critical for development and/or molting of the 0th instar. Because of this difficulty of incubation, perfect and circular incubation throughout the life cycle of sea skaters has not been possible.

The most apparent characteristic of the undescribed species inhabiting the off-shore area (50 km away from the shore) of Sumatra was the relative long body length in the last instar (5th instar and adult stage).

The characteristic of elongated body form may have developed on the way to evolutionary differentiation from other *Halobates* species relatively recently within the last 100 million years due to the famous Haeckel theory of phylogeny and ontogeny development [20], because the development of the body lengths of 0th to 3rd instars are similar to that of another similar-sized oceanic sea skater, *H. micans*.

## 5. Conclusions

Larvae and adults of four species of *Halobates* (*H. germanus*, *H. micans*, *H. micans* and undescribed species: *H.* sp.) were collected 50 km south-west of Sumatra. Adults of the undescribed species were characterized by a gourd-shaped body that was more than 5 mm long and longer than the other oceanic sea skaters we collected. There was a negative correlation between the amount of precipitation 19 h before collection and the number of *Halobates* specimens collected with a neuston net. The lower number of sea skaters collected could be associated with a drop in salinity and/or lower temperatures attributed to rain squalls.

## Figures and Tables

**Figure 3:**
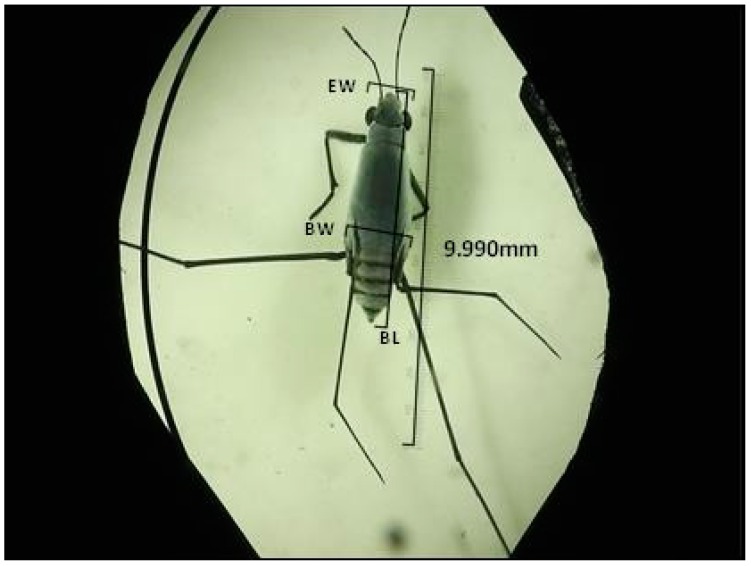
Adult female of *Halobates* sp. EW: Eye width, BW: Body width, BL: Body length, measured with a binocular microscope.

**Table 1:** A comparison of population density of oceanic sea skaters, *Halobates* among four areas of the open Indian and Pacific Oceans. Samplings were performed during seven cruises including the cruise for this study.

**A. MR15-04: Eastern Tropical Indian Ocean (this cruise)**								
1. 06°56′–06°58′ S 102°53′–102°54′ E (Station 1)								
	Total		*H. m.*	*H. g.*	*H. s.*	*H. p.*	*H.* sp.	AS ^#^
	Larvae	Adults						
Number	29	17	0	46	0	0	0	0.00744055
Density	3897.6	2284.8	0	6182.3	0	0	0	−
2. 04°02′–04°06′ S 101°52–101°55′ E (Stations 2–9)								
	Total		*H. m.*	*H. g.*	*H. s.*	*H. p.*	*H.* sp.	AS ^#^
	Larvae	Adults						−
Number	358	355	23	621	0	1	68	0.03072667
Density	11,651.1	11,553.5	748.5	20,210.5	0	32.5	2,213.1	−
**B. KH-07-04-Leg 1: Eastern Tropical Indian Ocean, 8°00′ N–6°35′ S, 86°00′ E–76°36′ E [7,8]**								
	Total		*H. m.*	*H. g.*	*H. s.*	*H. p.*	*H.* sp.	AS ^#^
	Larvae	Adults						
Number	1219	706	1886	111	0	0	0	0.044292
Density	29,147.5	15,939.7	42,581.1	2,506.1	0	0	0	−
**C. MR11-07-Leg 1: Eastern Tropical Indian Ocean, 1°55′ S, 83°24′ E; 8°00′ S, 80°30′ E) [9]**								
	Total		*H. m.*	*H. g.*	*H. s.*	*H. p.*	*H.* sp.	AS ^#^
	Larvae	Adults						−
Number	551	255	697	109	0	0	0	0.0438607
Density	12,562.5	5,813.9	15,891.2	2,485.1	0	0	0	0
**D. MR12-05-Leg 1 (Stations 1, 2 and 3): Western Subtropical and Tropical Pacific Ocean [5]**								
	Total		*H. m.*	*H. g.*	*H. s.*	*H. p.*	*H.* sp.	AS ^#^
	Larvae	Adults						
1. 13°59′ N 149°16′ E								
Number	44	73	43	0	74	0	0	0.0061659
Density	7136.0	11,839.3	6973.8	0	12,001.5	0	0	−
2. 1°55′ N 150°31′ E								
Number	66	379	8	437	0	0	0	0.0043914
Density	15,029.4	86,305.1	1821.7	99,512.7	0	0	0	−
3. 26°55′ S 165°34′ E								
Number	71	183	0	0	254	0	0	0.0066742
Density	10,638.0	27,419.0	0	0	38,057.0	0	0	−
**E. MR13-03 (Stations 1–10): Western Subtropical and Tropical Pacific Ocean [10]**								
	Total		*H. m.*	*H. g.*	*H. s.*	*H. p.*	*H.* sp.	AS ^#^
	Larvae	Adults						
1. 24°00′ N 138°10′ E (Station 1)								
Number	179	126	6	0	299	0	0	0.0031594
Density	56,656.5	39,881.1	1899.1	0	94,638.5	0	0	−
2. 1°55′ N 150°31′ E								
Number	484	119	276	327	0	0	0	0.02802519
Density	17,270.2	4246.2	9848.3	11,688.1	0	0	0	−
**F. KH-14-02 (Stations A and B): Western Subtropical and Tropical Pacific Ocean [11]**								
	Total		*H. m.*	*H. g.*	*H. s.*	*H. p.*	*H.* sp.	AS ^#^
	Larvae	Adults						
1. Northern Station at 47°00′ N 160°00′ N								
Number	0	0	0	0	0	0	0	0.0126451
Density	0	0	0	0	0	0	0	−
2. Southern Station at 25°00′ N 160°00′ E								
Number	593	254	0	847	0	0	0	0.0162708
Density	36,445.7	15,610.8	0	52,056.4	0	0	0	−
**G. MR14-06 leg 2: Western Tropical Pacific Ocean (10°00′ N–05°00′ S 130°00′ E–160°00′ E) [12]**								
	Total		*H. m.*	*H. g.*	*H. s.*	*H. p.*	*H.* sp.	AS ^#^
	Larvae	Adults						
Number	266	367	112	521	0	0	0	0.03036016
Density	8,761.5	12,088.2	3,689.0	17,160.6	0	0	0	−

*H. m.*: *Halobates micans*; *H. g.*: *Halobates germanus*; *H. s.*: *Halobates sericeus*; *H. p.*: *Halobates princeps*; *H.* sp.: undescribed species collected during this cruise. Density: number of individuals per km^2^; AS ^#^: Area of the surface swept by the neuston net (km^2^).

**Table 2:** Number of oceanic sea skaters, *Halobates* collected at locations from the tropical Indian Ocean from 20 November to 14 December 2015 during the science cruise, MR15-04 (N: Total number of individuals collected; *H. g.*: *Halobates germanus*, *H.* sp.: undescribed species, *H. p.*: *Halobates princeps*; Stat: station number; WT: water temperature (°C); AT: Air temp.; L: N of larvae; A: N of adults, E: N of exuviae; EG: number of eggs (on some substrates like as polystyrene form); Date: sampling date; Sampling was performed for 15 min. S: surface area which was swept by neuston net was expressed as value of flow-meter × 1.3 m of width of neuston net; WS: wind speed (m/s); W: weather; TD: time of day; WS: wind speed, CS: current speed (m/s) CD: current direction; F: female; M: male, No other species of oceanic sea skaters were collected in this area.

Latitude	Longitude	N	L	A		*H. g.*	*H.* sp.	*H. p.*	EG	E	Stat	WT	AT	WS	W	CS	Salinity (‰)	CD	TD	Date	S
				F	M																
**06°56′ S**	102°53′ E	7	4	1	2	7	0	0	0	0	St.1-1	28.7	28.9	10.3	Cloudy	1.0	31	151	19:22–19:37	20 November	1,991.0
**06°57′ S**	102°54′ E	17	11	5	1	17	0	0	0	0	St.1-2	28.7	28.9	8.9	Cloudy	1.0	31	145	19:45–20:00	20 November	1,929.5
**06°58′ S**	102°54′ E	22	14	4	4	22	0	0	0	1	St.1-3	28.7	28.9	11.2	Cloudy	1.1	31	141	20:02–20:17	20 November	1,803.0
**04°05′ S**	101°56′ E	14	9	3	2	5	9	0	0	0	St.2-1	29.9	28.2	5.9	R/C	0.7	28.9	122	19:16–19:31	23 November	1,955.0
**04°05′ S**	101°55′ E	9	6	1	2	1	8	0	0	0	St.2-2	29.9	28.2	5.3	Cloudy	0.6	28.9	115	19:36–19:51	23 November	1,754.0
**04°06′ S**	101°55′ E	8	5	3	0	1	7	0	0	0	St.2-3	29.9	28.2	6.3	Cloudy	0.6	28.9	119	19:56–20:11	23 November	1,712.0
**04°04′ S**	101°53′ E	10	6	2	2	10	0	0	0	0	St.3-1	29.3	28.2	6.3	Cloudy	0.4	30.0	219	19:12–19:27	26 November	964.5
**04°03′ S**	101°53′ E	6	3	2	1	6	0	0	0	0	St.3-2	29.3	28.2	5.5	Cloud	0.4	30.0	196	19:32–19:47	26 November	956.0
**04°02′ S**	101°53′ E	8	3	5	0	8	0	0	0	0	St.3-3	29.3	28.2	5.4	Cloudy	0.4	30.0	190	19:53–20:08	26 November	891.5
**04°04′ S**	101°53′ E	39	19	9	11	39	0	0	0	0	St.4-1	29.3	29.5	5.7	Cloudy	0.2	28.5	242	19:08–19:23	29 November	1,831.0
**04°05′ S**	101°53′ E	27	16	9	2	27	0	0	0	0	St.4-2	29.3	29.5	4.4	Cloudy	0.1	28.5	227	19:28–19:43	29 November	1,822.0
**04°05′ S**	101°52′ E	13	6	3	4	13	0	0	0	0	St.4-3	29.3	29.5	3.9	Cloudy	0.1	28.5	265	19:48–20:03	29 November	1,693.0
**04°03′ S**	101′53′ E	16	3	9	4	16	0	0	0	0	St.5-1	29.6	28.8	3.3	Cloudy	0.1	28.9	66	19:41–19:56	2 December	799.0
**04°03′ S**	101°53′ E	30	18	3	9	30	0	0	0	0	St.5-2	29.6	28.8	1.2	Cloudy	0.0	28.9	105	20:05–20:20	2 December	733.0
**04°03′ S**	101°53′ E	14	1	6	7	13	0	1	0	0	St.5-3	29.6	28.8	3.4	Cloudy	0.1	28.9	136	20:25–20:40	2 December	784.0
**04°04′ S**	101°53′ E	37	24	7	6	37	0	0	0	6	St.6-1	29	28.2	3.5	Cloudy	0.4	30.1	132	19:34–19:49	5 December	634.0
**04°03′ S**	101°53′ E	30	18	8	4	28	2	0	0	27	St.6-2	29	28.2	3.9	Cloudy	0.4	30.1	125	19:55–20:10	5 December	596.5
**04°03′ S**	101°52′ E	46	33	10	3	46	0	0	0	17	St.6-3	29	28.2	2.6	Cloudy	0.3	30.1	129	20:15–20:30	5 December	612.8
**04°04′ S**	101°53′ E	90	34	25	31	74	16	0	0	1	St.7-1	29.7	28.7	3.7	Cloudy	0.7	27.6	140	19:14–19:29	8 December	467.5
**04°04′ S**	101°53′ E	131	55	44	32	116	15	0	0	0	St.7-2	29.7	28.7	3.4	Cloudy	0.7	27.6	136	19:33–19:48	8 December	485.1
**04°05′ S**	101°54′ E	109	63	24	22	71	38	0	0	0	St.7-3	29.7	28.7	3.1	Cloudy	0.7	27.6	136	19: 52–20:07	8 December	466.0
**04°04′ S**	101°53′ E	2	0	2	0	2	0	0	0	0	St.8-1	30.3	30.0	2.9	Cloudy	0.3	27.7	126	19:07–19:22	11 December	725.0
**04°04′ S**	101°52′ E	4	4	0	0	3	1	0	1	0	St.8-2	30.3	30.0	2.2	Cloudy	0.3	27.7	117	19:26–19:41	11 December	816.0
**04°04′ S**	101°52′ E	6	3	2	1	5	1	0	3 (*H.* sp.)	0	St.8-3	30.3	30.0	3.9	Cloudy	0.3	27.7	107	19:46–20:01	11 December	762.5
**04°03′S**	101°53′ E	31	14	11	6	20	11 (*H. m*.)	0	0	0	St.9-1	29.2	27.7	4.0	Rainy	0.6	31.0	119	19:05–19:20	14 December	794.0
**04°02′S**	101°53′ E	23	12	6	5	17	6 (*H. m*.)	0	0	0	St.9-2	29.2	27.7	4.6	Rainy	0.5	31.0	122	19:26–19:41	14 December	710.5
**04°02′S**	101°53′ E	1 0	3	4	3	4	6 (*H. m*.)	0	1	0	St.9-3	29.2	27.7	5.1	Rainy	0.4	31.0	120	19:46–20:01	14 December	671.0

**Table 3:** Body size, body length and head width of larvae and adults of sea skaters collected in the sea area (50 km south-west) near Sumatra during the cruise MR15-04. (numerals in the table should be multiplied 33.3 μm; (number)).

***Halobates germanus* (Figure 1)**								
	**0th**	**1st**	**2nd**	**3rd**	**4th**	**5th**	**Adults**	
							**Females**	**Males**
Body length	20.35 ± 4.17	32.58 ± 4.87	42.67 ± 5.71	55.89 ± 7.85	72.67 ± 9.87	99.52 ± 7.70	113.34 ± 6.95	117.70 ± 4.41
	(17)	(62)	(38)	(35)	(35)	(63)	(176)	(140)
Body width	11.44 ± 2.96	17.97 ± 2.90	22.91 ± 2.88	28.76 ± 4.05	35.97 ± 4.27	48.16 ± 3.59	60.66 ± 2.30	52.58 ± 1.69
	(16)	(62)	(38)	(35)	(36)	(64)	(173)	(141)
Head width	8.25 ± 1.38	13.36 ± 2.00	17.53 ± 2.36	22.36 ± 3.25	28.49 ± 3.16	36.43 ± 1.49	41.28 ± 1.15	40.44 ± 1.07
	(16)	(62)	(38)	(35)	(36)	(64)	(173)	(141)
***Halobates* sp (Figure 2 and Figure 3)**								
	**0th**	**1st**	**2nd**	**3rd**	**4th**	**5th**	**Adults**	
							**Females**	**Females**
Body length	24.43 ± 2.66	35.53 ± 5.72	48.81 ± 5.19	68.19 ± 6.04	89.82 ± 16.11	142.36 ± 10.73	151.20 ± 17.15	161.75 ± 14.97
	(20)	(23)	(13)	(9)	(11)	(11)	(5)	(4)
Body width	11.40 ± 1.42	18.15 ± 3.95	27.08 ± 2.85	31.33 ± 2.35	37.46 ± 5.75	53.00 ± 6.69	67.00 ± 3.16	60.25 ± 9.54
	(20)	(23)	(13)	(9)	(11)	(11)	(5)	(4)
Head width	8.24 ± 1.44	14.15 ± 2.74	20.69 ± 2.18	27.56 ± 1.63	32.41 ± 3.61	42.27 ± 5.02	46.80 ± 0.57	46.63 ± 2.63
	(20)	(23)	(13)	(9)	(11)	(11)	(5)	(4)
***Halobates micans***								
	**0th**	**1st**	**2nd**	**3rd**	**4th**	**5th**	**Adults**	
							**Females**	**Males**
Body length	29.00 ± 8.49	33.50 ± 5.96	59.67 ± 7.64	72.70 ± 2.59	-	113.21 ± 16.49	114.50 ± 8.43	121.25 ± 10.84
	(2)	(4)	(3)	(5)		(17)	(11)	(6)
Body width	14.50 ± 7.78	17.25 ± 4.72	29.00 ± 1.73	42.00 ± 2.74	-	52.36 ± 4.40	65.36 ± 2.25	60.67 ± 1.21
	(2)	(4)	(3)	(5)		(17)	(11)	(6)
Head width	10.75 ± 6.01	13.25 ± 3.59	24.67 ± 1.53	31.50 ± 1.00	-	40.29 ± 1.52	43.18 ± 0.78	42.50 ± 1.48
	(2)	(4)	(3)	(5)		(17)	(11)	(6)
***Halobates princeps***								
	**0th**	**1st**	**2nd**	**3rd**	**4th**	**5th**	**Adults**	
							**Females**	**Females**
Body length	-	-	-	-	-	-	-	191.00
								(1)
Body width	-	-	-	-	-	-	-	70.00
								(1)
Head width	-	-	-	-	-	-	-	55.00
								(1)

**Table 4:** Statistical analysis on body length, body width and head width of sea skaters collected in the area near Sumatra in November–December 2015.

***ANCOVA (effects of species with covariance of stages)***									
	**Body length**			**Body Width**			**Head Widt**		
df	3			3			3		
*F*	93.312			29.003			84.787		
*p*	<0.001 ***			<0.001 ***			<0.001 ***		
***ANOVA (effects of species in each stage)***									
**A. Body length**									
	**Larvae**							**Adults**	
	**0th**	**1th**	**2th**		**3th**	**4th**	**5th**	**Females**	**Males**
df	2	2	2		2	1	2	2	2
*F*	*8.590*	*2.75*	*16.093*		*19.059*	*18.323*	*105.947*	*63.291*	*157.468*
*p*	0.001	0.069	<0.0001 ***		<0.001 ***	<0.001 ***	<0.001 ***	<0.001 ***	<0.001 ***
**B. Body width**									
	**Larvae**							**Adults**	
	**0th**	**1th**	**2th**		**3th**	**4th**	**5th**	**Females**	**Males**
df	2	2	2		2	1	2	2	2
*F*	*1.370*	*0.131*	*14.916*		*28.111*	*0.826*	*8.835*	*37.540*	*62.841*
*p*	*0.877*	*<0.001* ***	*<0.001* ***		*<0.001* ***	*0.369*	*<0.001* ***	*<0.001* ***	*<0.001* ***
**C. Head width**									
	**Larvae**							**Adults**	
	**0th**	**1th**	**2th**		**3th**	**4th**	**5th**	**Females**	**Males**
df	2	2	2		2	1	2	2	32000
*F*	2.001	1.069	20.134		29.104	12.083	36.868	71.326	95.374

*: 0.01 < *p* < 0.05; **: 0.001 < *p* < 0.01; ***: *p* < 0.001.

**Table 5:** Statistical analysis on body length, body width and head width between the two large-sized species of sea skaters, *Halobates micans* and *H.* sp. collected in the area near Sumatra in November–December 2015.

**A. Body Length**								
	**Larvae**						**Adults**	
	**0th**	**1th**	**2th**	**3th**	**4th**	**5th**	**Females**	**Males**
*z*	−0.804	−0.581	−2.225	−2.002	-	−2.993	−3.118	−2.566
*p*	0.441	0.561	0.026 *	0.045 *	-	0.003 **	0.002 **	0.010 *
**B. Body Width**								
	**Larvae**						**Adults**	
	**0th**	**1th**	**2th**	**3th**	**4th**	**5th**	**Females**	**Males**
*z*	−0.058	−0.206	−1.290	−3.003	-	−1.003	−0.668	−1.320
*p*	−0.954	0.837	0.239	0.003 **	-	0.316	0.491	0.187
**C. Head Width**								
	**Larvae**						**Adults**	
	**0th**	**1th**	**2th**	**3th**	**4th**	**5th**	**Females**	**Males**
*z*	−0.230	−0.582	−2.173	−2.809	-	−1.408	−3.189	−2.158
*p*	0.866	0.561	0.030 *	0.002 **	-	0.159	<0.001 ***	0.031*

*: 0.01 < p < 0.05; **: 0.001 < p < 0.01; ***: p < 0.001.
